# Classical Swine Fever Virus Infection Induces Endoplasmic Reticulum Stress-Mediated Autophagy to Sustain Viral Replication *in vivo* and *in vitro*

**DOI:** 10.3389/fmicb.2019.02545

**Published:** 2019-11-12

**Authors:** Erpeng Zhu, Wenxian Chen, Yuwei Qin, Shengming Ma, Shuangqi Fan, Keke Wu, Wenhui Li, Jindai Fan, Lin Yi, Hongxing Ding, Jinding Chen, Mingqiu Zhao

**Affiliations:** Department of Microbiology and Immunology, College of Veterinary Medicine, South China Agricultural University, Guangzhou, China

**Keywords:** classical swine fever virus, endoplasmic reticulum stress, unfolded protein response, autophagy, replication

## Abstract

Endoplasmic reticulum (ER) stress-mediated autophagy plays significant roles in replication and pathogenesis of many animal viruses. However, the relationship between ER stress, autophagy, and viral replication during *in vivo* and *in vitro* infection of classical swine fever virus (CSFV) remains unclear. In this study, we established a pig model for CSFV infection and found that viral loads of CSFV differed in 10 kinds of infected organs, and that the degree of tissue lesions was to some extent positively correlated with CSFV replication *in vivo*. Next, we found that CSFV infection not only induced ER stress and subsequently activated three unfolded protein responses (UPR) pathways including protein kinase R-like ER kinase (PERK), inositol requiring enzyme 1 (IRE1), and activating transcription factor-6 (ATF-6) pathways, but also triggered complete autophagy in main immune organs and partial nonimmune organs exhibiting severer pathological injuries and higher viral loads. However, only the IRE1 pathway and no autophagy were activated in some other nonimmune organs with slighter pathologies and lower viral loads. These results indicate a potential link between CSFV-induced ER stress and autophagy, both of which are associated with the CSFV replication *in vivo*. We further performed *in vitro* experiments and found that CSFV infection activates the PERK and IRE1 pathways and autophagy in cultured porcine kidney cell lines (PK-15) and macrophage cell lines (3D4/2), and pharmacological regulation of ER stress remarkably changed autophagic activities induced by CSFV, suggesting that CSFV-induced autophagy can be mediated by ER stress possibly *via* the PERK and IRE1 pathway. Furthermore, treatment with ER stress regulators significantly altered copy numbers of *NS5B* genes, expression of Npro proteins, and viral titers in CSFV-infected cells or in cells treated with autophagy regulators prior to CSFV infection, suggesting the requirement of ER stress-mediated autophagy for CSFV replication *in vitro*. Collectively, our data demonstrate that CSFV induces ER stress-mediated autophagy to sustain its replication *in vivo* and *in vitro*, which may be one of the potential strategies exploited by CSFV for immune evasion. This finding will provide new insights into mechanisms of replication and pathogenesis of CSFV, and development of new strategies for controlling CSF.

## Introduction

Classical swine fever (CSF), caused by classical swine fever virus (CSFV), is a worldwide and highly contagious disease of pigs that is notifiable to the World Organization for Animal Health (OIE) (Mehlhorn, 2015). As a member of the *Pestivirus* genus in the *Flaviviridae* family, CSFV contains an approximate 12.3-kb single-stranded sense RNA (ss(+)RNA) genome, encoding four structural proteins (C, Erns, E1, and E2) and eight non-structural proteins (Npro, P7, NS2, NS3, NS4A, NS4B, NS5A, and NS5B) (Tautz et al., 1999; Lefkowitz et al., 2018). Acute infection of CSFV causes persistent high fever, hemorrhages in multiple organs, and neurological, respiratory, and gastrointestinal symptoms in pigs with a very high mortality rate, leading to huge economic losses to the pig industry worldwide (Moennig, 1992; Lohse et al., 2012). Although clinical vaccinations can effectively prevent the outbreaks of CSF, there are no therapeutic drugs currently available on the market. CSFV replicates in leukocytes, especially in mononuclear macrophages, causing structural injuries and functional disorders in immune organs and subsequent immunosuppression in the diseased pigs (Floegel-Niesmann et al., 2003; Ji et al., 2015). Many viruses have evolutionarily developed specific or nonspecific strategies to evade host immune responses, during which many biological processes mediating the interplay between virus and host can be used for maintaining effective replication, infection, and pathogenesis of viruses (Pei et al., 2014; Peacock et al., 2017; Ying et al., 2018). However, mechanisms involved in CSFV replication and pathogenesis still need further investigations.

Endoplasmic reticulum (ER) is an important membranous organelle in eukaryotic cells. Homeostasis of the ER is a guarantee of maintaining normal cell activities. When the cells are exposed to stimuli including hypoxia, calcium overload, and free radical attack, dysfunctions of the ER occur and lead to accumulation of the misfolded/unfolded proteins in the lumen of ER and the imbalance of calcium homeostasis, resulting in ER stress (To Sing et al., 2015; Cybulsky, 2017). The ER responds to the burden of ER stress by activating a set of intracellular signaling pathways, known as the unfolded protein response (UPR), to restore normal function of the ER. There are three branches of the UPR: protein kinase R-like ER kinase (PERK), inositol requiring enzyme 1 (IRE1), and activating transcription factor-6 (ATF-6), which are not independent and together constitute a complex signaling network (Ron and Walter, 2007; Cybulsky, 2017). Under normal physiological conditions, glucose-regulated protein 78 (GRP78) binds to the three sensor proteins and inhibits their activities. Upon ER stress, GRP78 dissociates from the sensors and binds to the unfolded/misfolded proteins, and the released sensors are activated and initiate the following signaling pathways to alleviate ER stress through increasing protein-folding capacity of the ER, inhibiting global protein synthesis, and enhancing the ER-associated protein degradation (ERAD) of misfolded/unfolded proteins (Cybulsky, 2017). All these reactions aim to relieve the burden of ER and if they fail, they can trigger cellular dysfunction and eventually lead to cell death (Forman et al., 2003). In the past few years, the essential roles of the UPR have been implicated in many mammalian diseases, especially in viral diseases. Induction of ER stress and activation of the UPR signaling are general host responses to flavivirus and coronavirus infection, because replication of these viruses is closely associated with ER-derived membranes, and large amounts of viral proteins inevitably disturb the ER homeostasis and cause ER stress (Lin et al., 2002; Ambrose and Mackenzie, 2011; Peña and Harris, 2011; Fung and Liu, 2014). Our previous studies have revealed that CSFV infection induces the ER stress in the cultured porcine kidney PK-15 cells, benefiting its replication by activating the IRE1 pathway (He et al., 2017). However, the relationship between CSFV infection *in vivo* and ER stress-driven UPR, and the underlying mechanisms remain unclear.

Autophagy is an evolutionarily conserved cellular degradation and recycling process in eukaryotic cells, which contains three major types: microautophagy, macroautophagy (hereafter referred to as “autophagy”), and chaperone-mediated autophagy. Of these three types, autophagy is the best studied autophagic process (Deretic and Klionsky, 2018). During autophagic process, cytoplasmic components are sequestered into *de novo*-synthesized double-membrane vesicles termed autophagosomes, and then the formed autophagosomes fuse with the lysosomes for degradation and eventual recycling of the endocytosed macromolecules (Parzych and Klionsky, 2013; Deretic and Klionsky, 2018). Generally, basic autophagy occurs in normal cells at a low level, nevertheless, when the cells undergo adverse conditions, such as stress, infection, and cancer, autophagy can be further enhanced to degrade cytoplasmic macromolecules into metabolites for the recycling process in these cells, allowing for cell survival (Yorimitsu and Klionsky, 2005). Although autophagy functions as a pro-survival process in most cases by relieving the cells from various stress conditions (Gao et al., 2017), it also can be exploited as an potential immune escape strategy by some viruses, including hepatitis C virus (HCV), dengue virus (DENV), and CSFV (Pei et al., 2014; Dash et al., 2016; Wu et al., 2016). Furthermore, excessive or uncontrolled autophagy even can trigger a form of non-apoptotic cell death, termed “autosis,” which is dependent on the activity of Na^+^/K^+^-ATPase and has unique morphological features (Liu et al., 2013; Liu and Levine, 2015). Accordingly, dysregulated autophagy has been implicated in numerous human pathologies, including cancer, neurodegenerative diseases, and aging (Levine, 2007; Sayak et al., 2014; Onorati et al., 2017).

Autophagy is tightly regulated by several cellular signaling pathways, two typical pathways involved in nutrient starvation-induced autophagy are negatively regulated by the cAMP-dependent protein kinase A (PKA) and mammalian target of rapamycin (mTOR) pathways, respectively (Stephan et al., 2010). Besides, the ER stress-mediated autophagy has been studied extensively for years, and the complicated crosstalk between ER stress and autophagy has been proposed (Ron and Walter, 2007; Senft and Ronai, 2015). Evidences have revealed that many stimuli-induced ER stress can trigger autophagy either through the Ca^2+^-mediated AMPK signaling or through the UPR signaling (Yoshiyasu et al., 2013; Zaouali et al., 2013; Rashid et al., 2015). All the three UPR pathways of PERK, ATF-6, and IRE1 have been demonstrated to participate in autophagy induction, however, which UPR pathway is activated to mediate the induction of autophagy, and whether the cytoprotective autophagy or the autophagic cell death is induced in response to the ER stress, probably depend on the extent and type of stress, as well as the type of cells (Xing et al., 2007; He and Klionsky, 2009). Therefore, the molecular mechanisms involved in ER stress-mediated autophagy and the relationship between ER stress-mediated autophagy and diseases, still need further investigations. Our previous studies have demonstrated that CSFV-induced autophagy is beneficial to its replication, which is probably mediated by the suppression of reactive oxygen species (ROS)-dependent apoptosis in the cultured cell lines (Pei et al., 2014, 2016). However, the relationship between ER stress, autophagy, and viral replication during *in vivo* and *in vitro* infection of CSFV still needs to be clarified.

In the present study, basing on the established pig model for CSFV infection *in vivo*, we found that CSFV-induced ER stress and autophagy probably correlate with CSFV replication *in vivo*. *In vitro* experiments further confirmed that CSFV-induced autophagy can be mediated by ER stress, and ER stress-mediated autophagy functions as a replication strategy for sustaining CSFV infection *in vitro*. Taken together, our data demonstrate that CSFV induces ER stress-mediated autophagy to sustain its replication *in vivo* and *in vitro*, which may be one of the potential mechanisms exploited by CSFV for immune escape.

## Materials and Methods

### Cells, Virus, and Animal

The porcine kidney cell lines PK-15 (ATCC, CCL-33) and porcine macrophage cell lines 3D4/2 (ATCC, CRL-2845) were respectively cultured in Dulbecco’s modified Eagle medium (DMEM, Gbico, C11995500BT) and Roswell Park Memorial Institute (RPMI) 1640 medium (Gbico, C11875500BT) supplemented with 10% (v/v) fetal bovine serum (FBS, Gbico, 10091148) and 1% (v/v) penicillin-streptomycin (Gibco, 15140122). The cells were grown at 37°C in a humidified incubator (Thermo Fisher Scientific, HERAcell 150i) with 5% CO_2_. The CSFV-Shimen strain used in this study was our laboratory stock and was propagated in PK-15 cells. The virus titers were detected by 50% tissue culture infectious doses (TCID_50_) assays on PK-15 and 3D4/2 cells, and the multiplicity of infection (MOI) was calculated based on the obtained virus titers and the number of cells per well when seeded. Healthy 2-month-old pigs were purchased from Daguang farming Co., Ltd. and were pre-tested without CSFV, porcine reproductive, and respiratory syndrome (PRRSV), pseudorabies virus (PRV), and porcine parvovirus (PPV) infection. All pigs from mock group and CSFV infection group were separately housed in isolators with filtered air of positive pressure in a SPF animal facility in the laboratory animal center of South China Agricultural University.

### Plasmid, Reagents, and Antibodies

A recombinant plasmid pMD18-T-NS5B was our laboratory stock. Thapsigargin (TG, Abcam, ab120286) and Rapamycin (RAPA, Cell Signaling, 9904), Tauroursodeoxycholic acid (TUDCA, Millipore, 580549), 4-Phenylbutyric acid (4-PBA, Sigma, P21005), and 3-methyladenine (3-MA, Sigma, M9281) were respectively dissolved in dimethyl sulfoxide (DMSO) and sterile ultrapure water as stock solutions and further diluted with culture medium to the desired concentrations. The primary antibodies used in the study were specific for GRP78 (Santa Cruz, sc-13968), phosphor-IRE1 (S724) (Abcam, ab48187), ATF-6 (ABclonal, A0202), phospho-PERK (Thr980) (Cell Signaling, 3179), phospho-eIF2α (S51) (Bioworld, BS4787), eIF2α (Bioworld, BS3651), ATF-4 (ImmunoWay, YT1102), CHOP (Santa Cruz, sc-166682), LC3B (Cell Signaling, 2775), ATG5 (Novus Biologicals, NB110-53818), Beclin1 (Cell Signaling, 3495), SQSTM1/p62 (Cell Signaling, 39749), CSFV-E2 (MEDIAN/JBT, 9011), CSFV-Npro (kindly gift from Professor Xinglong Yu, Hunan Agricultural University, China), and Tubulin (Beyotime, AT819). Alexa Fluor 488-labeled goat anti-mouse IgG(H + L) (A0428), horseradish peroxidase (HRP)-labeled goat anti-rabbit IgG(H + L) (A0208), and goat anti-mouse IgG(H + L) (A0216) were purchased from Beyotime Biotechnology.

### Establishment of the Pig Model for Classical Swine Fever Virus Infection *in vivo*

Ten healthy 2-month-old pigs used in our study were randomly divided into two groups: a mock group and a CSFV infection group, five pigs in each group. About 1 × 10^5^ TCID_50_/ml viral stock was intramuscularly injected into bilateral neck of pigs for CSFV infection (Luo et al., 2017; Gou et al., 2017a), and the mock-infected pigs were only injected with the same volume of phosphate-buffered saline (PBS). Anal temperature of pigs from each group was measured everyday till 7 days post-infection (dpi) by a mercury thermometer. Clinical symptoms of the experimental pigs were observed and recorded daily, and clinical score (CS) values were calculated as described by Mittelholzer et al. (2000) to evaluate the situation of CSFV infection. Briefly, the calculation of CS values was obtained by evaluating 10 CSF clinical signs, including liveliness, body tension, body shape, breathing, walking, skin, eyes/conjunctiva, appetite, defecation, and leftovers in feeding trough. Each parameter was judged as either normal (score 0), slightly altered (score 1), distinct clinical sign (score 2), or severe CSF symptom (score 3). Scores from these parameters were assessed and totalized daily (maximum score: 30). This system for judging virulence based on the CS (CS > 15 and fever>41°C as a highly virulent strain; 15 ≥ CS >5 and 41°C ≥ fever ≥ 40°C as a moderately virulent strain; CS ≤ 2 and fever<40°C as a low or avirulent strain) has been used by many laboratories (Mittelholzer et al., 2000; Floegel-Niesmann et al., 2003; Everett et al., 2010; Luo et al., 2017). Pigs were sacrificed at 7 dpi, and pathological changes of immune organs (tonsil, thymus, spleen, and inguinal lymph node) and non-immune organs (heart, lung, liver, kidney, brain, and intestine) were observed and analyzed. To confirm the infection of CSFV, *E2* structural genes and E2 proteins in the collected immune organs (tonsil, thymus, spleen, and inguinal lymph node) were detected by reverse transcription (RT)-PCR and immunohistochemistry (IHC) assays, respectively. In our study, three pigs randomly selected from each group were sacrificed according to animal welfare and then subject to the following tests and analyses, at the meantime, these pigs and the rest pigs were altogether used in other omics studies performed by our team.

### Viral Infection and Biochemical Intervention

*In vitro* experiments were performed to further confirm the inference proposed *in vivo*. For CSFV infection, PK-15 or 3D4/2 cells were grown to approximately 80% confluence in 6-well cell culture plates and then infected with 1 MOI of CSFV (0.5 MOI of CSFV for replication studies) or mock-infected with same volume of medium. After a 1.5 hour (h) absorption at 37°C, the inoculum was removed and the cells were washed twice with PBS and further incubated in maintenance media (DMEM or 1640 containing 2% FBS) at 37°C for different times until harvesting of the cells. For biochemical intervention, cells were pretreated with ER stress or autophagy regulators for the indicated hours, specifically, autophagy inducer RAPA (working concentration: 100 nM) for 1 h, autophagy inhibitor 3-MA (5 mM) for 4 h, ER stress agonist TG, ER stress inhibitors TUDCA and 4-PBA for 6 h at the indicated concentrations, respectively. After a 1.5 h absorption of CSFV, the cells were further cultured in maintenance media in the presence (TUDCA, 4-PBA, RAPA, and 3-MA) or absence (TG) of the drugs for 24 h. In addition, cells pretreated the same amount of dimethyl sulfoxide (DMSO, Sigma, D2650) or sterile ultrapure water were regarded as the control group. Samples of each treatment were collected at the indicated time points for real-time RT-PCR, Western blot, and or virus titration assays.

### RT-PCR and Real-Time RT-PCR

The status of CSFV infection *in vivo* was detected by CSFV-specific RT-PCR assays targeting the *E2* genes or by real-time RT-PCR assays targeting the CSFV-*NS5B* genes, and CSFV infection in cells was detected by real-time RT-PCR or western blot assays using a rabbit anti-Npro antibody. Briefly, 100 mg of tissues from each pig or one-well cells from 6-well microplates in each treatment were collected for extraction of total RNA using TRIzol reagent (Invitrogen, 15596026) according to the manufacturer’s instructions, and 1 μg of each RNA was subsequently reversely transcribed into cDNA with a 5×All-In-One RT MasterMix (with AccuRT Genomic DNA Removal Kit) (Applied Biological Materials, G492), and then diluted with a 1:4 ratio in sterile ddH_2_O for following assays. For RT-PCR, 2 μl of the resultant cDNA was used as the template to amplify CSFV-*E2* genes as described previously (He et al., 2017). The expected product was identified by 1% agarose gel electrophoresis. The viral loads in each tissue and cell samples were determined by absolute quantification of CSFV *NS5B* gene copies through real-time RT-PCR. For real-time RT-PCR, 2 μl of the resultant cDNA was used as the template to amplify CSFV-*NS5B* genes as previously described (He et al., 2017). A recombinant plasmid pMD18-T-*NS5B* was used as standard samples, and the standard curve was constructed using 10-fold serially diluted standard plasmids. Real-time RT-PCR was performed on a CFX Connect™ Real Time PCR Detection System (Bio-Rad, 1855200). Each template was run in triplicate. The viral loads in terms of *NS5B* copies /g tissues or *NS5B* copies/mL were determined using the linear regression plot generated by GraphPad Prism 6 software. The effect of drug treatments on mRNA expression of ER stress and autophagy indicators, including *GRP78*, *ATG5*, *ATG12*, and *Beclin1*, was also detected by real-time RT-PCR as previously described (He et al., 2017). Relative mRNA expression of the target genes were assessed using the 2^−ΔΔCT^ method and normalized to the housekeeping *Glyceraldehyde-3-phosphate dehydrogenase* (*GAPDH*) gene. The primers used in this study are indicated in Table 1.

**Table 1:** Primer information for reverse transcription (RT)-PCR and real-time RT-PCR.

Gene	Sequence(5′–3′)	GenBank	Size (bp)
CSFV-E2^*^	Forward: CCACCTGGAAAGAATACAGC Reverse: CTCGTATCAAACGGGCAC	AF092448.2	241
CSFV*-NS5B*	Forward: CCTGAGGACCAAACACATGT Reverse: TGGTGGAAGTTGGTTGTGTCTG	AY775178.2	174
*GRP78*	Forward: CCTACTCGTGCGTTGGGGT Reverse: GACGGCGTGATGCGGTT	XM_001927795.7	79
*ATG5*	Forward: GGTTTGAATATGAAGGCACACCA Reverse: TGTGATGTTCCAAGGAAGAGCTG	AM087014.1	98
*ATG12*	Forward: CTTCTTCCGCTTCAGTTTCC Reverse: TGTGTCTCCCACAGCCTTTA	NM_001190282.1	92
*Beclin1*	Forward: AGGAGCTGCCGTTGTACTGTTCT Reverse: TGCTGCACACAGTCCAGGAA	NM_001044530.1	94
*GAPDH*	Forward: TGGAGTCCACTGGTGTCTTCAC Reverse: TTCACGCCCATCACAAACA	NM_001206359.1	121

* *Primers of CSFV-*E2* were used for reverse transcription (RT)-PCR, and all the rest primers were used for real-time RT-PCR*.

### Pathological Examination and Immunohistochemistry

The experimental pigs were sacrificed at 7 dpi for pathological examination and immunohistochemistry. Macroscopic examination was performed immediately after sacrifice of the experimental pigs. Macroscopic photographs of the indicated immune organs and non-immune organs from each pig were taken for pathological analysis. For microscopic examination, the indicated tissues were fixed in 4% paraformaldehyde and then subjected to preparation of pathological sections with a routine protocol. Briefly, the fixed tissues were flushed with running water overnight, after dehydration in a graded ethanol series, the tissues were clarified with fresh xylene and then embedded into paraffins. Serial sections of 4 μm thickness were prepared, followed by Hematoxylin and Eosin (HE) staining. For further confirmation of CSFV infection, immunohistochemical assays exploiting a mouse anti-CSFV E2 monoclonal antibody were performed as previously described (Gou et al., 2017b). The Dako REAL EnVision Detection Systems (DAKO, K500711-2) was used to display the staining. After counterstaining with hematoxylin for 3 min, sections were dehydrated and then subjected to microscopic imaging with a NIKON Eclipse Ci biological microscope (Japan) under a magnification of 200×. For each tissue, three visual fields from three sections were randomly selected for further analyses. The appreciable brown staining is considered as positive expression of E2 proteins.

### SDS-PAGE and Western Blot

For preparation of tissue samples, 100 mg of the indicated tissues from each pig were cut into pieces and added into 200 μl of RIPA lysis buffer (Beyotime, P0013B) containing 4 μl protease and phosphatase inhibitor cocktail (50×) (Beyotime, P1050), and the mixture was smashed in a tissue homogenizer (Monad, GS60101) and then incubated on ice for 20 min. For preparation of cell samples, one-well cells from 6-well microplates in each treatment were collected and lysed on ice for 20 min with 150 μl of RIPA lysis buffer containing protease and phosphatase inhibitors. After centrifugation of lysates at 13,000 r/min for 15 min at 4°C, the supernatant was collected for quantitation of the solubilized protein using a BCA protein assay kit (Beyotime, P0012). Equal amounts of samples were mixed with 5× SDS-PAGE loading buffer and boiled for 10 min, and then separated on 10 or 12.5% polyacrylamide gel electrophoresis (PAGE) gels and then transferred onto 0.22 μm polyvinylidene fluoride (PVDF) membranes (Bio-Rad, 3010040001). The membranes were then blocked for 1.5 h at 37°C with 5% (m/v) non-fat dry milk or 5% bovine serum albumin in PBS with 0.1% (v/v) Tween 20 (PBST) and then incubated at 4°C overnight with proper dilution of primary antibodies against LC3B, p62, Beclin 1, ATG5, p-PERK, p-eIF2α, eIF2α, ATF-4, CHOP, ATF-6, p-IRE1, GRP78 and tubulin, respectively. After three washes with PBST, the membranes were further incubated for 1 h at 37°C with HRP-conjugated goat anti-rabbit or goat anti-mouse immunoglobulin antibodies diluted 1:2000 in PBST. After three washes with PBST, the protein bands were visualized using an ECL detection kit (Beyotime, P0018) in a Tanon Fine-do ×6 chemiluminescent imaging system (Tanon), and signal quantification was achieved using ImageJ software.

### Virus Titration

To further investigate the effect of ER stress-mediated autophagy on viral yields, cells were treated as described above in the “biochemical intervention,” and the cells and supernatants of each treatment were collected for rapid freezing and thawing for three times, and then subjected to virus titration determined by TCID_50_ assays on PK-15 and 3D4/2 cells as described previously (Pei et al., 2014). Briefly, cells cultured in 96-well microplates were inoculated with 10-fold serially diluted virus for a 1.5 h incubation at 37°C, and further incubated at 37°C for 48 h. Cells were fixed with pre-cooled absolute ethanol at −20°C for 30 min, and viral proteins were visualized by an indirect immunofluorescence assay using mouse anti-CSFV E2 antibody and Alexa Fluor 488-labeled goat anti-mouse IgG(H + L) secondary antibody. Virus titers were calculated according to Reed-Muench method (Reed and Muench, 1938) and expressed as Log_10_ (TCID_50_/ml).

### Cell Viability Assays

Cell viability was detected by the CCK-8 assays. Briefly, cells were seeded in 96-well microplates (1 × 10^4^ cells/well) and cultured at 37°C for 24 h. After removal of the culture medium, the cells were treated with vehicle or the indicated concentrations of ER stress or autophagy regulators diluted in maintenance media for the indicated time according to the requirements of the different experiments. Next, cells were further incubated with the CCK-8 (Dojindo, CK04) diluted in fresh medium (final concentration of 10%, v/v) for 1 h at 37°C, and then the absorbance was measured at 450 nm with a Ledetect 96 microplate reader (LABEXIM PRODUCTS). Each sample was assayed in triplicate and the results expressed as a percentage of the control group were averages from two independent experiments.

### Statistical Analysis

The data in the study are expressed as the mean ± standard deviation (SD) and were analyzed by one-way ANOVA or two-way ANOVA using the GraphPad prism 6 software. *p* less than 0.05 were considered statistically significant.

## Results

### Establishment of the Pig Model for Classical Swine Fever Virus Infection *in vivo*

**Figure 1:**
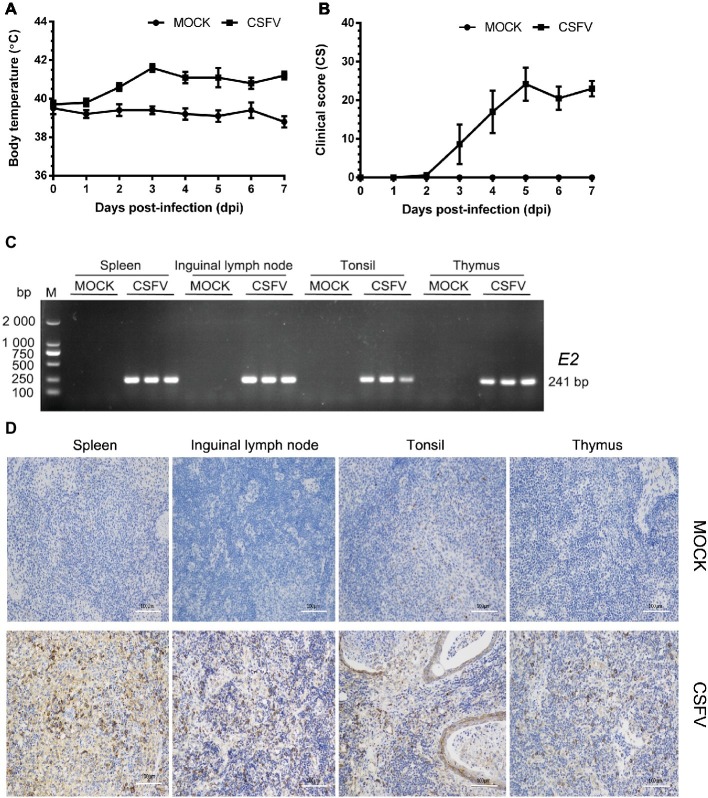
Establishment of a pig model for CSFV infection *in vivo*. Ten healthy 2-month-old pigs with CSFV and CSFV antibody negative were randomly divided into two groups: a mock group and a CSFV infection group. About 1 × 10^5^ TCID_50_/ml viral stock was intramuscularly injected into bilateral neck of pigs for CSFV infection, and pigs in the mock group were only injected the same volume of PBS. Body temperature was measured everyday till to 7 dpi **(A)**, and clinical symptoms of the experimental pigs were observed and recorded daily for calculation of CS values **(B)**. All the experimental pigs were sacrificed at 7 dpi for samples collections, and three pigs from each group were randomly selected for the following tests and analyses. **(C)** For further confirmation of CSFV infection, *E2* structural genes in the collected immune organs (tonsil, thymus, spleen and inguinal lymph node) were detected by RT-PCR. The expected product (241 bp) was identified by 1% agarose gel electrophoresis. **(D)** Simultaneously, expression of E2 proteins in these organs were analyzed by IHC method exploiting a mouse anti-E2 monoclonal antibodies. Pictures were captured with a NIKON Eclipse Ci biological microscope (Japan) under a magnification of 200×. The appreciable brown staining is considered as positive expression of E2 proteins. Bar, 100 μm.

**Figure 2:**
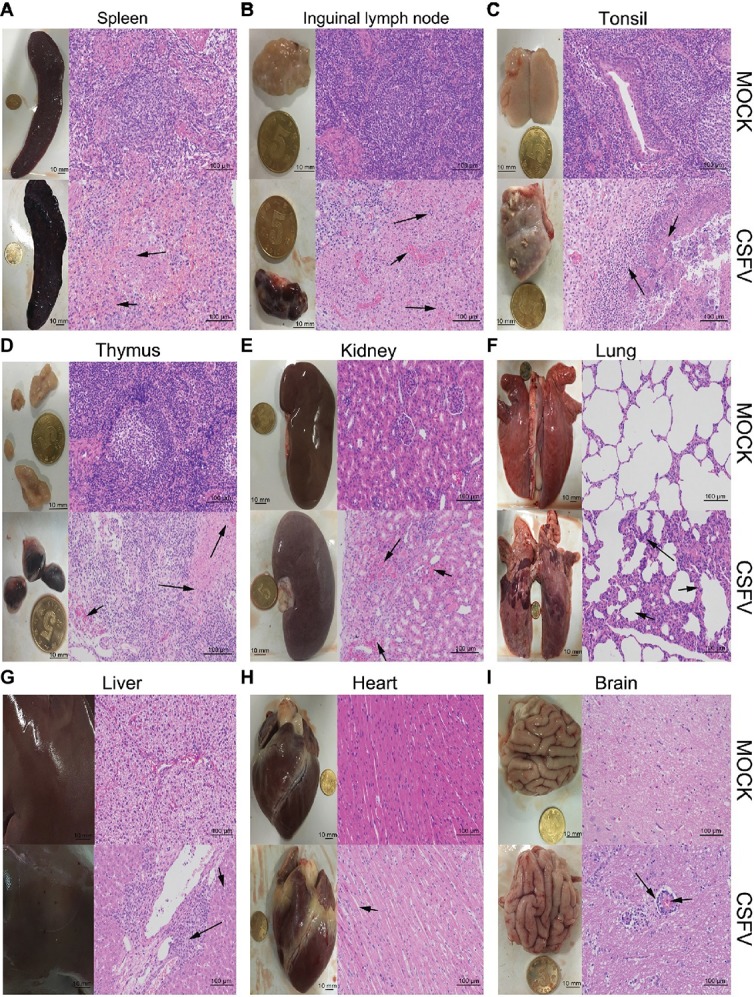
Macroscopic and microscopic examination of organs from the mock- and CSFV-infected pigs. The experimental pigs were sacrificed at 7 dpi and the indicated organs were collected for pathological analyses. Macroscopic examination was performed immediately after sacrifice of the experimental pigs; bar: 10 mm. For microscopic examination, tissue samples were subjected to preparation of tissue sections and Hematoxylin and Eosin (HE) staining, and then histopathological changes were analyzed with a NIKON Eclipse Ci biological microscope (Japan) under a magnification of 200×. Bar: 100 μm. In each panel, the upper left and upper left respectively show gross and microscopic lesions from mock-infected pigs, in which no pathological changes were observed; the lower left and lower right respectively show gross and microscopic lesions from pigs infected with CSFV, and varying degrees of hemorrhagic necrotizing inflammation could be seen in several organs. Histopathological changes as indicated by black arrows with different lengths can be seen in the panels. **(A)** Spleen, **(B)** inguinal lymph node, **(D)** thymus: short arrows indicate hemorrhage, and long arrows show lymphocyte necrosis and disintegration, accompanied with infiltration of inflammatory cells. **(C)** Tonsil: short arrows indicate flaky necrosis of lymphocytes, and long arrows show inflammatory cell infiltration around the necrotic foci. **(E)** Kidney: short arrows indicate severe hemorrhage in the superficial cortex, and long arrows show a large amount of inflammatory cell infiltration in the mesenchyma. Alveolar atrophy and alveolar wall thickening (short arrow) as well as inflammatory cell infiltration (long arrow) can be seen in infected lung **(F)**. Hepatocyte steatosis (short arrow) and interstitial cell hyperplasia with concomitant inflammatory cell infiltration (long arrow) in the portal area were observed in infected liver **(G)**. No obvious histopathological changes were obtained in infected heart, except for slight swelling of cardiomyocytes **(H)**. Occasionally, vascular sleeve-like changes can be seen in cerebral parenchyma **(I)**.

In this study, we firstly established a pig model of CSFV infection *in vivo* by monitoring changes in body temperature, clinical symptoms and pathology, as well as CSFV infection. During the test, the average body temperature of pigs in the mock group was maintained between 38.3 and 39.9°C. The average body temperature of CSFV-infected pigs increased to the top (41.6°C) at 3 dpi, and thereafter remained at 40.8–41.2°C, displaying a high fever retention state (Figure 1A). Typical clinical characteristics of CSF including high fever, diarrhea and neurological symptoms emerged at 3 dpi and lasted till to 7 dpi. And then we scored the clinical symptoms of the experimental pigs as described by Mittelholzer et al. (2000). The average peak clinical score (CS) value of CSFV-infected pigs gradually increased and reached over 15 since 4 dpi (Figure 1B), which is in accordance with the criteria for virulent CSFV strains (peak CS > 15, T > 41.0°C) (Mittelholzer et al., 2000). The experimental pigs were sacrificed at 7 dpi for pathological examination and sample collections. Typical pathological changes of CSF were observed and analyzed (as shown in Figure 2). To further confirm the infection of CSFV, a pair of specific primers were designed for detection of CSFV *E2* genes in the collected immune organs (tonsil, thymus, spleen and inguinal lymph node) through reverse transcription (RT)-PCR method. Simultaneously, E2 expression in the collected immune organs was analyzed by IHC method exploiting a mouse anti-E2 monoclonal antibodies. The results of both RT-PCR (Figure 1C) and IHC (Figure 1D) showed that E2 expression was positive in immune organs from CSFV-infected pigs rather than mock-infected pigs. Collectively, these results demonstrated the successful establishment of *in vivo* pig model for CSFV infection, providing a solid guarantee for the following assays.

### Classical Swine Fever Virus Infection *in vivo* Causes Varying Degrees of Hemorrhagic Necrotizing Inflammation in Multiple Organs

The experimental pigs were sacrificed at 7 dpi for pathological examination. From the view of macroscopic pathology, immune organs from the CSFV-infected pigs presented very typical lesions, including hemorrhagic infarction around the edge of spleen, extensive hemorrhagic and necrotic foci in tonsil, thymus and superficial lymph nodes, as well as severe ulcer in tonsil; besides, different degrees of hemorrhage were found in other non-immune organs, such as lung, kidney, liver, brain, etc., typically, intensive tiny bleeding points were visible in the kidney, which is commonly named as the “freckle kidney” (Figures 2A–I, lower left panels). In contrast, no abnormal lesions were observed in the organs collected from the mock-infected pigs (Figures 2A–I, upper left panels). In terms of microscopic histopathology, CSFV infection caused severe hemorrhagic and necrotic inflammation in tonsils, spleen, thymus and lymph nodes, which were characterized by the infiltration of massive erythrocytes and inflammatory cells among the tissue cells and the extensive lymphocytes depletion and tissue necrosis (Figures 2A–D, lower right panels), and were basically in lines with typical histopathological changes of CSF. Besides, pulmonary hemorrhage, consolidation and interstitial pneumonia, renal hemorrhagic inflammation, tubular degeneration and necrosis were observed in the CSFV-infected pigs (Figures 2E,F, lower right panels). Although pictures of the injured intestines were not presented due to mechanical damage during preparation of tissue sections, intestinal villus shortening and exudative inflammation still can be observed (data not shown). As for other organs, slight hemorrhage and interstitial hepatitis, slight swelling of cardiomyocytes, as well as scarce vascular sleeve-like changes in cerebral parenchyma were observed in the CSFV-infected pigs (Figures 2G–I, lower right panels). By contrast, there were no histopathological changes observed in the mock-infected pigs (Figures 2A–I, upper right panels). Our results suggested that CSFV infection *in vivo* causes varying degrees of hemorrhagic necrotizing inflammation in several organs, and the severities of tissue injury in immune organs, kidney, lung and intestine are higher than those in other organs, probably resulting from the tissue tropism of CSFV infection *in vivo*.

### Tissue Loads of Classical Swine Fever Virus Are Associated With the Severity of Tissue Injury

**Figure 3:**
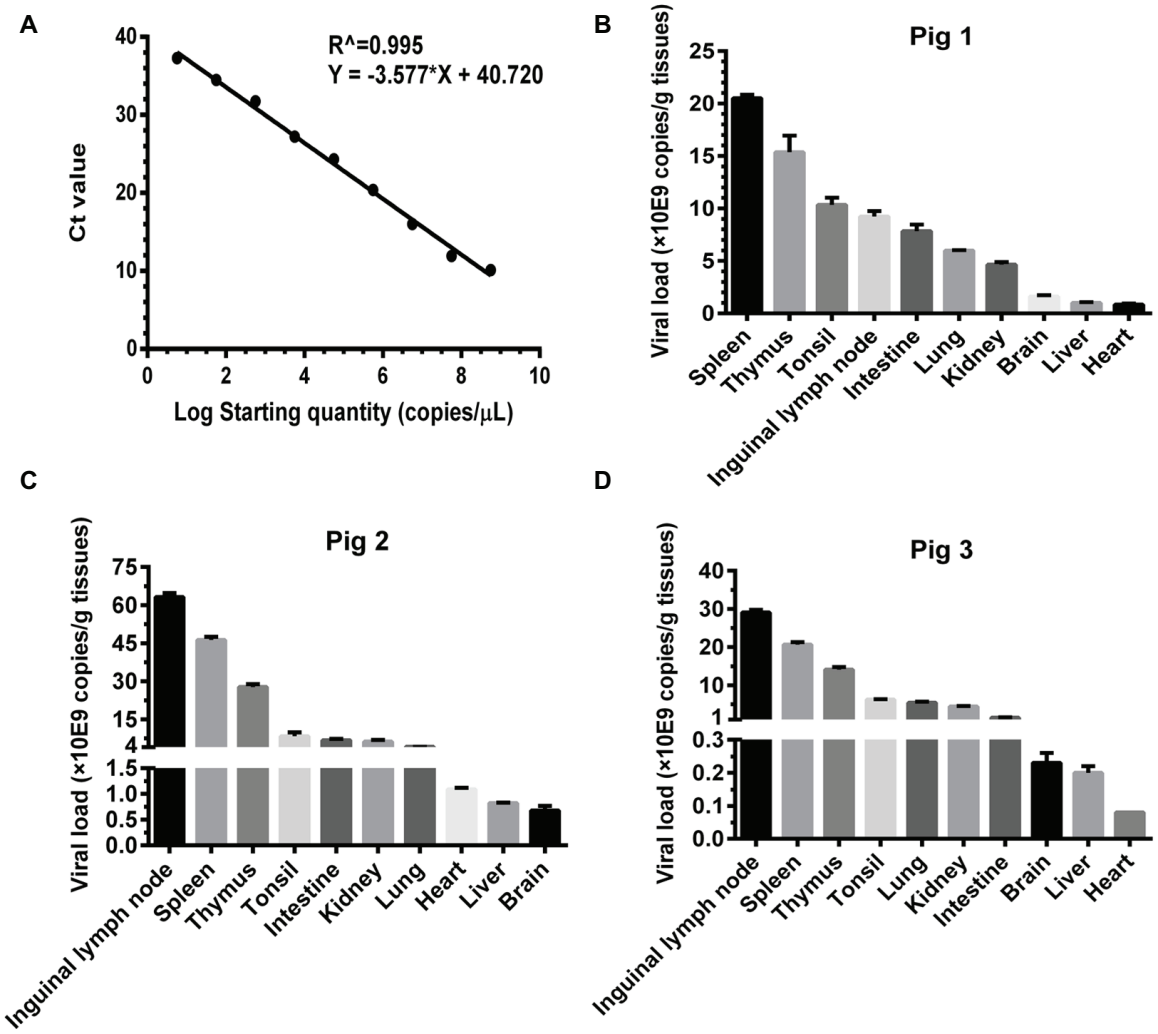
Viral loads of CSFV vary with the degree of injury in infected organs. About 100 mg tissues as indicated from the experimental pigs were collected for extraction of total RNA, 1 μg total RNA was transcribed into cDNA as template for absolute quantification of CSFV *NS5B* gene copies through real-time RT-PCR. A recombinant plasmid pMD18-T-*NS5B* was used as standard samples. The standard curve was constructed using 10-fold serially diluted standard plasmids. A linear regression plot was generated using GraphPad Prism 6 software (GraphPad Software Inc., San Diego, Calif). The slope, the Y-intercept and the R^2^ value were determined **(A)**. The viral loads in terms of *NS5B* copies/g tissues from the experimental pigs **(B–D)** were determined using the linear regression plot. Each template was ran in triplicate.

Basing on the established pig model, we determined the viral loads (CSFV *NS5B* copies/g tissues) in the infected organs by absolute quantitative RT-PCR. Firstly, a standard curve was constructed using 10-fold serially diluted standards, and data shown confirm that these assays are performed with high efficiencies (Figure 3A). The viral loads were determined using the linear regression plot. As we expected, there was no detectable expression of CSFV genes in organs collected from mock-infected pigs (data not shown), while viral genes were differentially detectable in these organs from CSFV-infected pigs. Viral loads in immune organs (tonsil, spleen, thymus, and lymph nodes), and partial non-immune organs (lung, kidney, and intestine) of the infected pigs were generally higher than those in heart, liver and brain (Figures 3B–D). Although individual differences existed in the three pigs randomly selected from CSFV group, it seemed that viral loads in organs with severe pathological changes were higher than those in organs with slight pathological changes. Anyway, the results above suggested that tissue viral titer of CSFV is positively correlated with the severity of tissue injury.

### Classical Swine Fever Virus Infection *in vivo* Causes Endoplasmic Reticulum Stress and Differentially Activates Unfolded Protein Responses Pathways in Infected Organs

**Figure 4:**
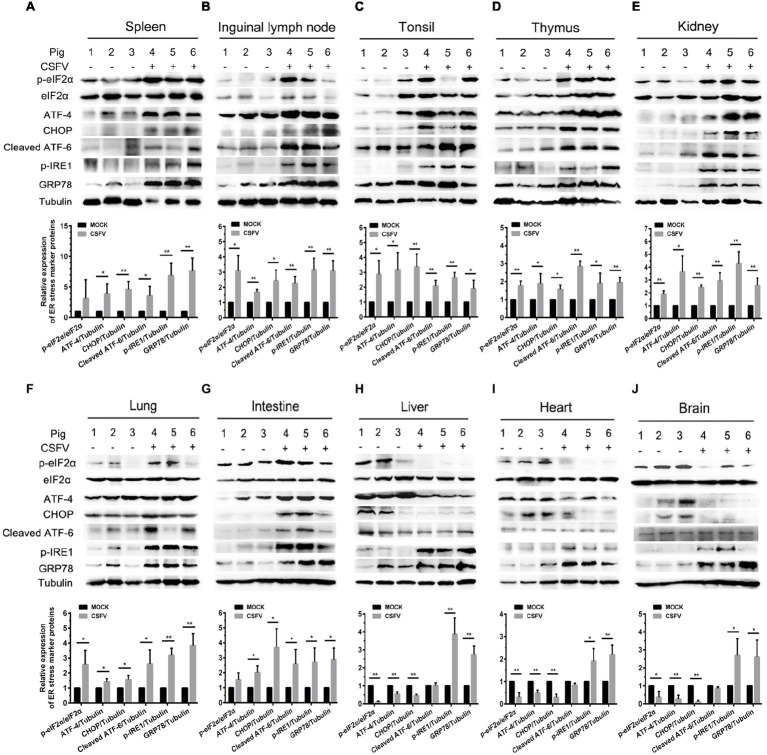
CSFV infection *in vivo* causes ER stress and differentially activates UPR pathways in infected organs. About 100 mg immune tissues **(A–D)** or non-immune tissues **(E–J)** as indicated from the experimental pigs were collected at 7 dpi for preparation of protein samples as described in “Materials and Methods,” samples were quantitated by BCA assays and then loaded for western blot analyses using specific antibody against p-eIF2α, total eIF2α, ATF-4, CHOP, cleaved ATF-6, p-IRE1, GRP78 and tubulin. Tubulin was used as a loading control. Grayscale value of the bands were analyzed with ImageJ software, and generations of images for protein quantitation and all statistical analyses were performed with GraphPad Prism 6. All the data shown are expressed as mean ± SD values of three independent experiments. Multiple *t* tests: ^*^p < 0.05; ^**^p < 0.01.

ER stress has been shown to be a potential strategy used by many viruses to regulate viral replication for effective infection (Rong et al., 2016; He et al., 2017; Lee et al., 2018). To clarify the relationship between CSFV infection *in vivo* and ER stress, we investigated the effect of CSFV infection on the expression of UPR-related molecules in the immune and non-immune organs collected from the experimental pigs by Western blot analyses. The status of CSFV infection has been demonstrated by real-time RT-PCR and IHC assays (Figures 1D, 3B–D). Here we found that CSFV infection caused a significant increase in GRP78 expression in all of the collected organs, suggesting the occurrence of ER stress. However, the degree of ER stress differs in infected organs. Specifically, all of the three UPR pathways (PERK, IRE1, and ATF-6) were activated in the immune organs (tonsil, spleen, thymus, and lymph nodes) and partial non-immune organs (lung, kidney, and intestine), as demonstrated by increasing protein expressions in phosphorylated (p)-IRE1, cleaved ATF-6, and p-PERK, as well as their downstream targets including GRP78, p-eIF2α, ATF-4, and CHOP (Figures 4A–G), suggesting the induction of a serious ER stress in these organs. However, only the IRE1 pathway was activated in the heart, liver, and brain (Figures 4H–J). Of note, the PERK pathways in these organs were significantly suppressed, demonstrated by the decreasing protein expression in downstream targets of PERK, including p-eIF2α, ATF-4, and CHOP. Besides, no obvious activation of ATF-6 pathway was obtained in these organs. Taken into account the above results, our results suggested that CSFV-induced ER stress appeared to be more serious in the organs with severer pathological changes and higher viral titers, while relatively mild ER stress was detectable in organs with slighter pathological changes and lower viral titers, which implied that ER stress-mediated UPR pathways are associated with CSFV replication *in vivo*.

### Classical Swine Fever Virus Infection *in vivo* Differentially Induces Autophagy in Infected Organs

**Figure 5:**
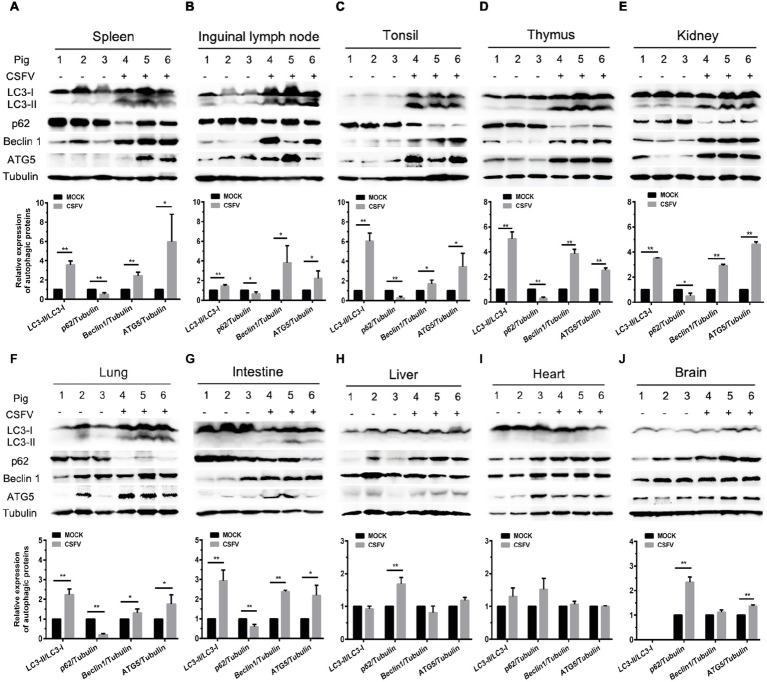
CSFV infection *in vivo* differentially induces autophagy in infected organs. 100 mg immune tissues **(A–D)** or non-immune tissues **(E–J)** as indicated from the experimental pigs were collected at 7 dpi for preparation of protein samples as described in the “materials and methods”, samples were quantitated by BCA assays and then loaded for western blot analyses using specific antibody against LC3B, p62, Beclin1, ATG5 and tubulin. Tubulin was used as a loading control. Grayscale value of the bands were analyzed with ImageJ software, and generations of images for protein quantitation and all statistical analyses were performed with GraphPad Prism 6. All the data shown are expressed as mean ± SD values of three independent experiments. Multiple *t* tests: ^*^p < 0.05; ^**^p < 0.01. Original blots for Figures 5D,E,G,H,J are provided in the Supplementary Material.

Autophagy is an evolutionarily conserved cellular degradation and recycling process in eukaryotic cells, and has been implicated in many diseases (Levine and Kroemer, 2008; Mizushima et al., 2008; Santanacodina et al., 2017). Many viruses have developed specific strategies to regulate autophagy for effective infection (Pei et al., 2014; Dash et al., 2016; Wu et al., 2016). Our previous study has revealed that CSFV-induced autophagy is beneficial to its replication *in vitro* (Pei et al., 2014), while the details *in vivo* and the underlying mechanism need to be further investigated. ER stress-driven UPR is one of the important pathways to regulate autophagy. To determine the relationship between CSFV-induced autophagy and ER stress during *in vivo* infection of CSFV, we further investigated the effect of CSFV infection on the expression of autophagic proteins in immune and non-immune organs from the experimental pigs by Western blot analyses. The results showed that CSFV infection caused significantly increase in ATG5, Beclin1 and LC3-II accumulations, with the concomitant decrease in autophagy substrate p62 in immune organs including spleen, inguinal lymph nodes, tonsils and thymus (Figures 5A–D), as well as in non-immune organs of lung, kidney and intestine (Figures 5E–G) from the CSFV-infected pigs, indicating the induction of complete autophagy in these organs. In contrast, no significant changes in ATG5, Beclin1 and LC3-II expression were detectable in the non-immune organs, including heart, liver and brain from CSFV-infected pigs (Figures 5H–J), indicating that no autophagy is induced in these organs, and that CSFV-induced activation of autophagy in pigs is to some extent tissue-specific. Taken into consideration the results of pathological changes and viral loads, as well as activation of UPRs, our results showed that CSFV infection *in vivo* induces serious ER stress and autophagy in the organs with severer pathological changes and higher viral titers, while relatively mild ER stress and no autophagy was detectable in organs with slighter pathological changes and lower viral titers, suggesting that CSFV-induced ER stress and autophagy are associated with the replication of CSFV in infected organs, and that CSFV-induced ER stress may be linked to autophagy, which needs to be further confirmed.

### Classical Swine Fever Virus Infection Induces Endoplasmic Reticulum Stress-Induced Autophagy in Cultured Porcine Cell Lines

**Figure 6:**
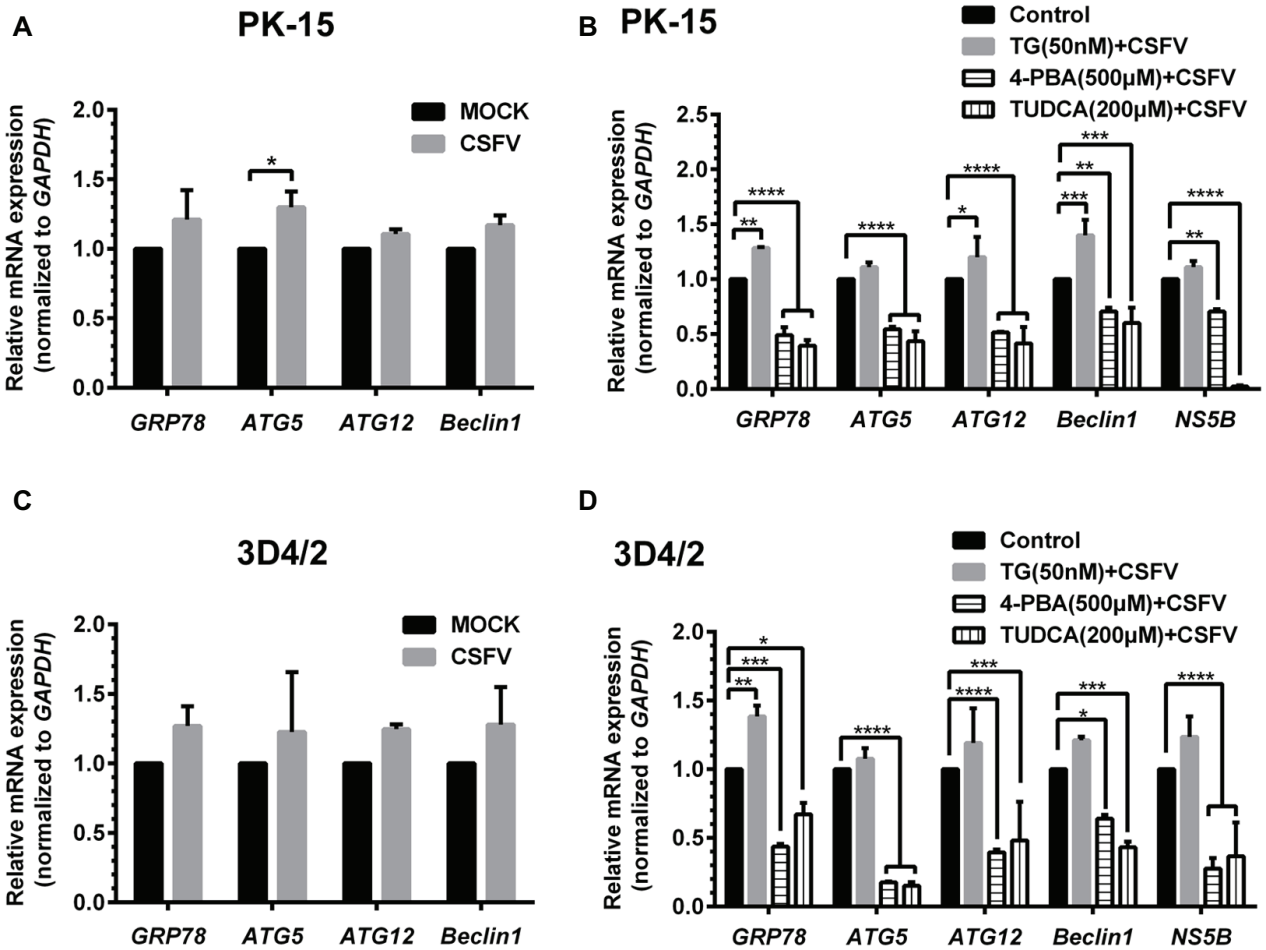
Treatment with ER stress regulators changes transcriptional levels of autophagic genes in CSFV-infected PK-15 and 3D4/2 cells. PK-15 **(A,B)** and 3D4/2 **(C,D)** cells cultured in 6-well plates were pretreated with 50 nM TG or 200 μM TUDCA or 500 μM 4-PBA for 6 h, and then subject to a 1.5 h of incubation with 1 MOI of CSFV. The cells were further cultured in maintenance media in the presence (TUDCA and 4-PBA) or absence (TG) of the drugs for 24 h. Additionally, cells pretreated with only DMSO (0.1%) were regarded as the control group. The CSFV group was only infected with 1 MOI of CSFV, and the MOCK group was only treated with the same amount of media. The cells of each treatment were collected for extraction of total RNA, and 1 μg total RNA from each sample was transcribed into cDNA as template for relative quantification of the indicated genes through real-time RT-PCR. Relative mRNA expression of the target genes were assessed using the 2^−ΔΔCT^ method and normalized to the *GAPDH* gene. Each template was ran in triplicate. The results are expressed as mean ± SD values of two independent experiments. Generations of images for quantitation and statistical analyses were performed with GraphPad Prism 6. All data presented were analyzed by two-way ANOVA tests: ^*^p < 0.05; ^**^p < 0.01; ^***^p < 0.001; ^****^p < 0.0001.

**Figure 7:**
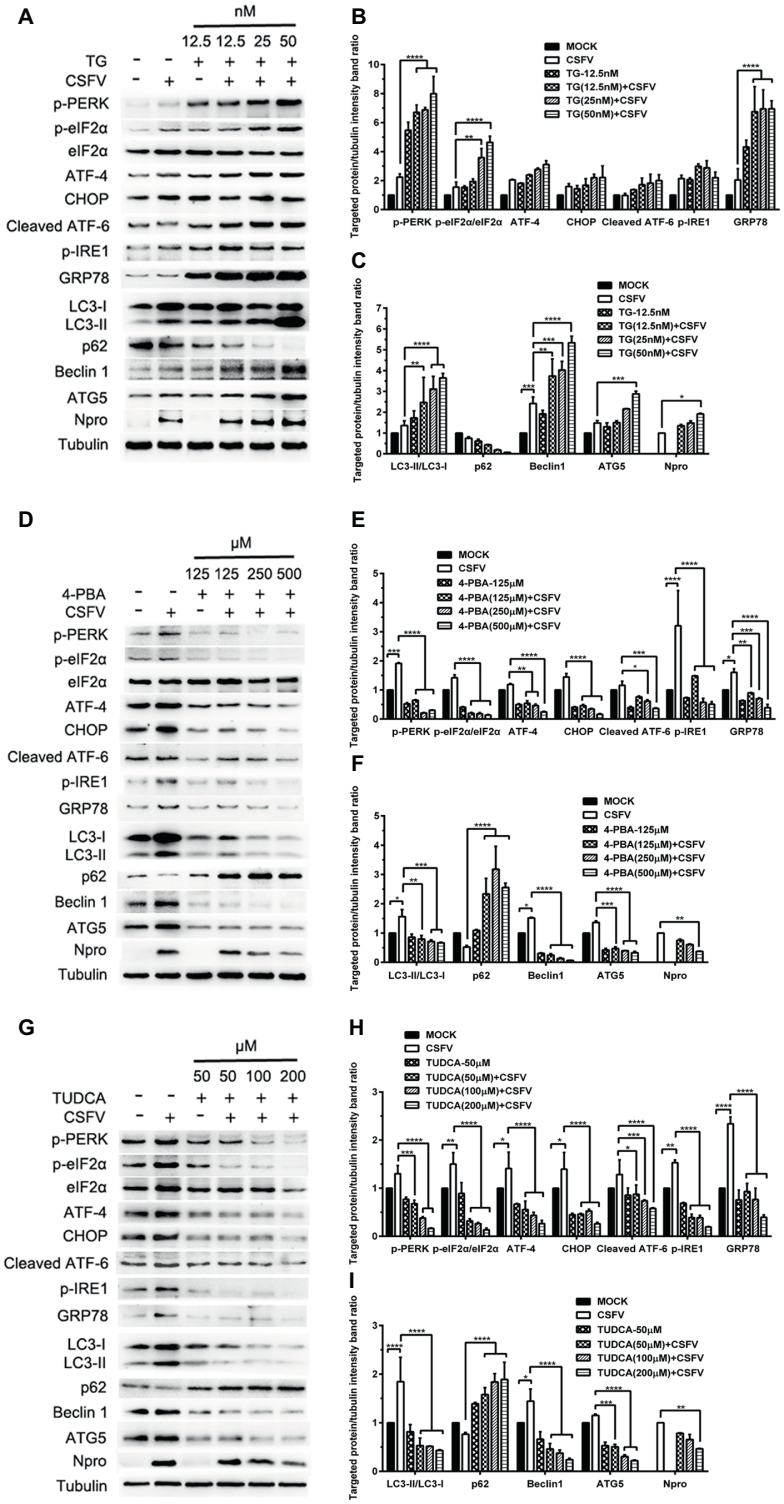
Pharmacological regulation of ER stress changes expression of autophagic proteins in CSFV-infected PK-15 cells. PK-15 cells cultured in 6-well microplates were pretreated with ER stress agonist TG (12.5, 25 and 50 nM) **(A)**, ER stress inhibitors 4-PBA (125, 250 and 500 μM) **(D)** and TUDCA (50, 100 and 200 μM) **(G)** for 6 h, respectively, and then subject to CSFV infection. After a 1.5 h of incubation, the cells were further cultured in maintenance media in the presence (TUDCA, 4-PBA) or absence (TG) of the drugs for 24 h. Additionally, cells pretreated with only MDRV or mock infection or only drugs were regarded as the control group. Cells were collected for preparation of protein samples in accordance with description in the “materials and methods”. Samples were quantitated by BCA assays and then loaded for western blot analyses using specific antibody against the indicated targets. Tubulin was used as a loading control. Grayscale value of the bands were analyzed with ImageJ software, and generations of images for protein quantitation and all statistical analyses **(B,C,E,F,H,I)** were performed with GraphPad Prism 6. All the pictures of western blot represent one of two independent experiments, and all the quantitative data shown are expressed as mean ± SD values of two independent experiments. Two-way ANOVA test: ^*^p < 0.05; ^**^p < 0.01; ^***^p < 0.001; ^****^p < 0.0001.

**Figure 8:**
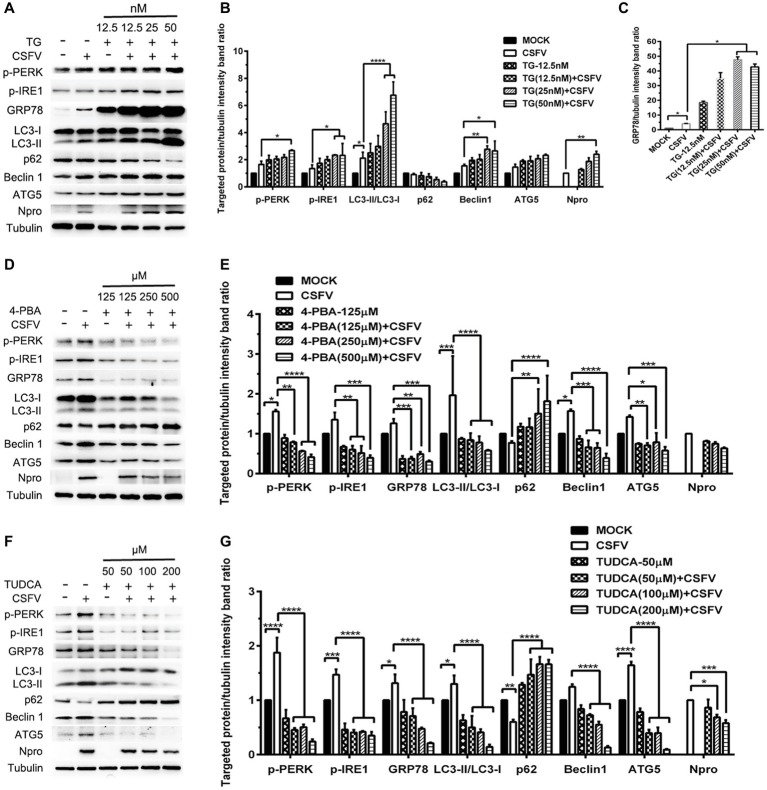
Pharmacological regulation of ER stress alters expression autophagic proteins in CSFV-infected 3D4/2 cells. 3D4/2 cells were treated with the indicated concentrations of TG **(A)**, 4-PBA **(D)** and TUDCA **(F)** prior to viral infection as described in the legend of Figure 6, and then collected for preparation of protein samples according to the description in the “Materials and Methods”. Samples were quantitated by BCA assays and then loaded for western blot analyses using specific antibody against the indicated targets. Tubulin was used as a loading control. Grayscale value of the bands were analyzed with ImageJ software, and generations of images for protein quantitation and all statistical analyses **(B,C,E,G)** were performed with GraphPad Prism 6. All the pictures of western blot represent one of two independent experiments, and all the quantitative data shown are expressed as mean ± SD values of two independent experiments. Two-way ANOVA test: ^*^p < 0.05; ^**^p < 0.01; ^***^p < 0.001; ^****^p < 0.0001.

ER stress-mediated autophagy has been well studied due to its significant roles in many diseases (Rashid et al., 2015; Senft and Ronai, 2015; Cybulsky, 2017). Having shown that there is a potential link between CSFV-induced ER stress and autophagy in infected pigs, we further performed *in vitro* experiments to confirm this relationship. The effect of pretreatment with ER stress agonists (TG) and inhibitors (4-PBA and TUDCA) on autophagic activities in the CSFV-infected PK-15 and 3D4/2 cells were respectively analyzed with real-time RT-PCR and Western blot assays. We found that CSFV infection not only caused ER stress and activated UPR pathways, but also induced autophagy at 24 hpi in cultured cells, as demonstrated by elevated transcriptional level of ER stress- and autophagy-related genes (*GRP78, ATG5, ATG12*, *and Beclin1*) (Figures 6A,C), as well as remarkable increase in protein expression of ER stress and autophagic indicators, such as GRP78 and LC3-II (Figures 7, 8). Treatment with TG, a potent inhibitor of Ca^2+^ transport ATPase to disturb ER homeostasis and cause ER stress, not only further increased CSFV-induced transcription of *GRP78*, *ATG12* and *Beclin1* gene (Figure 6B), but also remarkably enhanced CSFV-increased protein expression of ER stress indicators, including p-PERK, p-eIF2α and GRP78 (Figures 7A,B), and simultaneously significantly up-regulated expression of autophagy proteins, including LC3-II, Beclin1 and ATG5, in a dose-dependent manner (Figures 7A,C). A decreasing expression of p62 was also observed in TG-treated cells after CSFV infection (Figures 7A,C). The similar results were obtained in cultured 3D4/2 cells (Figures 6D, 8A–C). It suggested that upregulation of ER stress to some extent increases autophagic activities in CSFV-infected cells. In contrast, treatment with ER stress inhibitors 4-PBA and TUDCA markedly inhibited CSFV-induced ER stress in PK-15 cells represented by significant suppression of CSFV-increased *GRP78* mRNA level (Figure 6B) and decreasing protein expression of p-PERK and p-IRE1, as well as their downstream targets p-eIF2α, ATF-4, CHOP and GRP78 (Figures 7D,E,G,H), and meanwhile suppressed CSFV-increased transcriptional levels of *ATG5*, *ATG12* and *Beclin1* gene (Figure 6B), and inhibited protein expression of LC3-II, Beclin1 and ATG5 with concomitant increase of p62 expression in a dose-dependent manner (Figures 7D,F,G,I). The similar results were obtained in 3D4/2 cells (Figures 6D, 8D–G), indicating that downregulation of ER stress effectively inhibits autophagic activities in CSFV-infected cells, and that ER stress-activated PERK and IRE1 signalings are potentially required for CSFV-induced autophagy, further confirming our results obtained *in vivo*. Moreover, the status of CSFV infection was monstered by detecting expression of viral proteins Npro, and intriguingly, the expressions of Npro proteins were significantly increased by TG treatment, while were remarkably inhibited by treatment with 4-PBA and TUDCA in a dose-dependent manner (Figures 7A,D,G, 8A,D,F), implying that CSFV-induced ER stress probably affects viral replication in cultured cells, which was in line with our results in Figures 6B,D that ER stress regulators markedly changed copy numbers of CSFV-*NS5B* genes in both PK-15 and 3D4/2 cells. Taken together, our data suggested that CSFV infection induces ER stress-induced autophagy both *in vitro* and *in vivo*, which potentially regulates viral replication.

### Endoplasmic Reticulum Stress-Mediated Autophagy Is Beneficial to CSFV Replication in Cultured Cells

**Figure 9:**
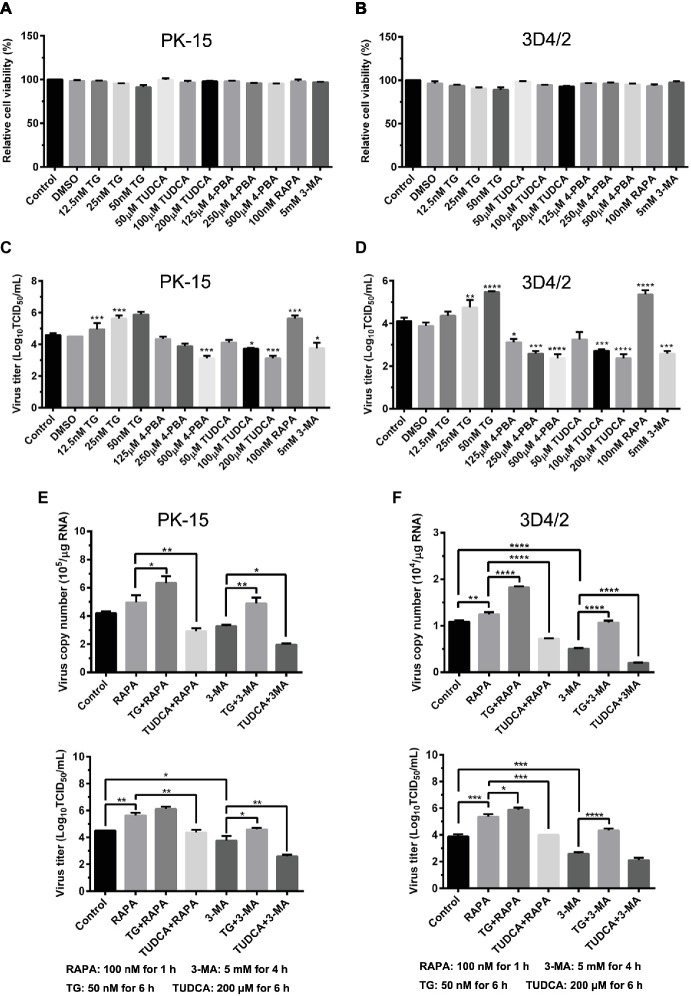
Autophagy-regulated CSFV replication in cultured PK-15 and 3D4/2 cells can be mediated by ER stress. **(A)** The cell viability of PK-15 and 3D4/2 **(B)** cells were measured by the CCK-8 assays after treatments with the indicated concentrations of TG (12.5, 25 and 50 nM), 4-PBA (125, 250 and 500 μM) and TUDCA (50, 100 and 200 μM). Cells pretreated with only DMSO (0.1%) or sterile ultrapure water were regarded as the control group. After further incubation for the indicated time as described in the “Materials and Methods”, cells were further incubated with 10% (v/v) CCK-8 diluted in fresh medium for 1 h at 37°C, followed by measurement of the absorbance at 450 nm with a Ledetect 96 microplate reader. Each sample was assayed in triplicate and the data represent the mean ± SD of two independent experiments. For virus titration assays, PK-15 **(C)** and 3D4/2 **(D)** cells cultured in 6-well microplates were pretreated with the indicated concentrations of TG, 4-PBA, RAPA and 3-MA for the indicated time, followed by infection with 0.5 MOI of CSFV. Twenty-four hours post-infection, the cells and supernatants of each treatment were collected for virus titration determined with TCID_50_ assays through an indirect immunofluorescence assay using mouse anti-CSFV E2 antibody and Alexa Fluor 488-labeled goat anti-mouse IgG(H + L) secondary antibody. Virus titers were calculated according to Reed-Muench method and expressed as Log_10_(TCID_50_/mL). Each sample was assayed in four replicates and the data represent the mean ± SD of two independent experiments. To investigate the effect of ER stress-mediated autophagy on CSFV replication *in vitro*, PK-15 **(E)** and 3D4/2 **(F)** cells cultured in 6-well plates were pretreated with 50 nM TG or 200 μM TUDCA for 6 h, and then subject to treatment with 100 nM RAPA for 1 h or 5 mM 3-MA for 4 h prior to CSFV infection. After a 1.5 h of incubation, the cells were further cultured in maintenance media in the presence (TUDCA, RAPA, 3-MA) or absence (TG) of the drugs for 24 h. Additionally, cells pretreated with only DMSO (0.1%) were regarded as the control group. The cells and supernatants of each treatment were collected for detecting the copies of *NS5B* genes and viral titers using real-time RT-PCR and TCID_50_ assays, respectively. The results are expressed as mean ± SD values of two independent experiments. For all assays, generations of images for quantitation and all statistical analyses were performed with GraphPad Prism 6. All data presented were analyzed by one-way ANOVA tests: ^*^p < 0.05; ^**^p < 0.01; ^***^p < 0.001; ^****^p < 0.0001.

Since we have showed that CSFV induces ER stress-mediated autophagy, both *in vivo* and *in vitro*, whether this process affects CSFV replication or not was further studied. The effect of ER stress or autophagy regulators on CSFV infection in PK-15 and 3D4/2 cells were analyzed with real-time RT-PCR and TCID_50_ assays. As shown in Figures 9A,B, CCK-8 assays showed that either ER stress or autophagy regulators at the indicated working concentrations have no significant influence on viabilities of both PK-15 or 3D4/2 cells. We have showed that treatment with ER regulators can effectively changes the expressions of Npro proteins in a dose-dependent manner (Figures 7A,D,G, 8A,D,F), implying that viral replication is regulated by CSFV-induced ER stress. Here we further found that treatment with ER stress agonists (TG) induces a dose-dependent increase in viral titers measured by TCID_50_ assays in CSFV-infected cells compared with the control group, however, viral titers were significantly inhibited by treatment with varying concentrations of ER stress inhibitors (4-PBA and TUDCA) (Figures 9C,D), confirming the requirement of ER stress in CSFV replication *in vitro*. We also found autophagy regulators remarkably altered both copies of *NS5B* genes and viral titers in cultured cells (Figures 9C–F). These results suggested that CSFV-induced ER stress and autophagy are beneficial to its replication, which is in line with our previous studies (Pei et al., 2014; He et al., 2017). Since we have showed above that ER stress is required for CSFV-induced autophagy and viral replication, ER stress-mediated autophagy can be a potential mechanism for regulating CSFV replication. To further confirm their relationship, PK-15 and 3D4/2 cells were or not pretreated with 50 nM TG or 200 μM TUDCA followed by treatment with autophagy regulators and infection with 0.5 MOI of CSFV, and then incubated in maintenance media in the presence of ER stress regulators for 24 h, and then cells were collected to determine the copies of *NS5B* genes and viral titers using real-time RT-PCR and TCID_50_ assays, respectively. Results showed that TG pretreatment further enhanced RAPA-increased viral replication, while TUDCA treatment significantly suppressed RAPA-increased viral replication in CSFV-infected cells compared with RAPA-treated cells, suggesting that viral replication enhanced by RAPA-induced autophagy can be regulated by ER stress (Figures 9C–F). Likewise, TUDCA treatment further reduced 3-MA-decreased viral replication, while TG pretreatment effectively elevated 3-MA-decreased viral replication, suggesting that viral replication reduced by 3MA-inhibited autophagy can also be regulated by ER stress (Figures 9C–F). The similar results were obtained in the PK-15 and 3D4/2 cells. Taken together, our results suggested that ER stress-mediated autophagy induced by CSFV functions as a replication strategy for sustaining CSFV infection.

## Discussion

CSFV replicates in leukocytes, especially in mononuclear macrophages, and CSFV infection in pigs induces apoptosis in varieties of immune cells, such as peripheral blood leukocytes, and lymphocytes in spleen and lymph nodes, ultimately leading to immunocyte depletion and immunosuppression (Summerfield et al., 1998; Floegel-Niesmann et al., 2003; Ji et al., 2015). However, no cytopathogenic effect is detectable in cultured cells infected with CSFV (Ruggli et al., 2005), making it difficult to fully understand the replication and pathogenesis of CSFV. Apart from apoptosis, several biological processes mediating the interplay between virus and host, such as ER stress and autophagy, have attracted numerous researchers’ focuses and have been implicated in the replication and pathogenesis of viruses (Lin et al., 2002; Dash et al., 2016; Wu et al., 2016; He et al., 2017). Our previous studies have revealed that CSFV infection induces ER stress and autophagy to facilitate viral replication *in vitro* (Pei et al., 2014; He et al., 2017), however, the details *in vivo* and the underlying mechanism remain to be further investigated. In the present study, we found that CSFV infection in pigs differentially induced ER stress and autophagy in several organs, which were to some extent tissue-specific and associated with the replication of CSFV. *In vitro* experiments further confirmed the ER stress-mediated autophagy as a potential strategy used by CSFV to sustain its replication, which suggests a possible immune escape mechanism of CSFV and will provide new insight into the mechanisms involved in replication and pathogenesis of CSFV.

To perform *in vivo* experiments, we first established a pig model for CSFV infection, providing a guarantee for the follow-up experiments. Histopathology analysis of the organs from the CSFV-infected pigs showed that CSFV infection caused varying degrees of hemorrhagic necrotizing inflammation and dramatic lymphocytes depletion in several organs, and that the severities of tissue injury in immune organs, kidney, lung and intestine were higher than those in other organs, which are similar with previous several studies associated with tissue tropism of CSFV (Liu et al., 2011; Sun et al., 2011). Tissue injuries of immune organs and massive reduction of lymphocytes caused by CSFV infection may be the main reason of immunosuppression in the diseased pigs. We further found the severities of tissue injury are positively correlated with tissue viral loads of CSFV. Although the differential tissue tropism and replication of CSFV in pigs have also been detected in some other studies (Liu et al., 2011; Sun et al., 2011), the underlying mechanisms responsible for this phenomenon are seldom investigated, which encourages us to explore potential factors responsible for differential replication of CSFV *in vivo*. Based on our previous studies, ER stress-mediated autophagy becomes our preference.

Perturbation of the ER homeostasis by various stimuli can trigger ER stress in eukaryotic cells. Then the cells evoke a highly conserved mechanism to alleviate ER stress and re-establish ER homeostasis by activating three UPR pathways: PERK, IRE1 and ATF-6 (Ron and Walter, 2007; Cybulsky, 2017). The activated PERK phosphorylates eIF2α and results in global translational attenuation to alleviate the immediate pressure on the ER. However, phosphorylation of eIF2α allows for preferential translation of ATF-4 to up-regulate the genes involved in restoring ER homeostasis (Renata and Reed, 2013). Upon activation, the phosphorylated IRE1 cleaves a 26-nucleotide intron from the X box-binding protein 1 (XBP1) mRNA to generate an active transcription factor XBP1(s), which up-regulates UPR genes encoding ER chaperones and components of the ER-associated degradation (ERAD) machinery to restore ER homeostasis (Renata and Reed, 2013; Li et al., 2015). Activated ATF-6 translocates to the Golgi apparatus for cleavation, and then the cleaved ATF-6 fragment translocates into the nucleus and activates the transcription of ER chaperones for promoting protein folding, transport, and ER expansion (Renata and Reed, 2013). Emerging evidences indicate that a great number of viruses can induce ER stress and differentially activate the three UPR pathways during their infection (Lin et al., 2002; Peña and Harris, 2011; Dash et al., 2016). To explore the potential factors responsible for different tissue viral titers during CSFV infection *in vivo*, we further studied the effect of CSFV infection on ER stress in pigs and found that CSFV infection induced ER stress and subsequently activated the PERK, IRE1 and ATF-6 pathways in main immune organs and partial nonimmune organs exhibiting severer pathological injuries and higher viral loads. The increasing expression in the activated sensor proteins (p-PERK, p-IRE1, and cleaved ATF-6) and their downstream targets (p-eIF2α, ATF-4, CHOP, and GRP78) probably alleviate CSFV-induced serious ER stress in pigs, attempting to provide a suitable condition for efficient virus replication. The UPR can adapt to changing severity and duration of ER stress and trigger apoptosis when the stress cannot be overcome. CHOP, an important targets driven by ATF-4, is involved in the apoptotic cell death under prolonged or strong ER stress (Renata and Reed, 2013; To Sing et al., 2015). Of note, the increasing expression in CHOP induced by CSFV may be associated with the elevated caspase-3 and PARP cleavation, together contributing to apoptotic cell death in organs suffering severe ER stress (data not shown). The remarkable lymphocyte reduction is very likely attributed to the ER stress-mediated apoptosis. Nevertheless, whether ER stress-mediated apoptosis is induced and its underlying mechanisms during CSFV infection need further confirmation. Unlike the organs mentioned above, only the IRE1 pathway was activated in partial nonimmune organs with slighter pathological changes and lower viral loads, and the ATF-6 and PERK pathways were inactive or even suppressed, suggesting a mild ER stress in these organs. Therefore, it is suggested that CSFV-induced ER stress and activation of UPR are probably associated with CSFV replication *in vivo*.

As an evolutionarily conserved cellular degradation and recycling process in eukaryotic cells, autophagy functions as a pro-survival mechanism in most cases by relieving the cells from adverse conditions, such as nutrient deprivation and pathogen infection (Lum et al., 2005; Xu et al., 2013), however, dysfunctional autophagy can trigger autophagic cell death (Liu and Levine, 2015). Autophagy has been implicated in numerous viral diseases and is to some degree virus-specific. Some viruses (herpes simplex virus type 1 and human immunodeficiency virus type 1) can inhibit autophagic activities to enhance viral replication (Orvedahl et al., 2007; Kyei et al., 2009), while some viruses (DENV, influenza A virus, HCV and Muscovy duck reovirus) induce autophagy as a strategy to facilitate replication (Ray et al., 2008; Marlène et al., 2009; Zhi et al., 2009; Wu et al., 2017). Basing on our previous finding that autophagy enhances the replication of CSFV in cultured cells (Pei et al., 2014), here we found that CSFV infection significantly increased the expression of autophagic markers in main immune organs and partial nonimmune organs exhibiting severer pathological injuries and higher viral loads, including LC3-II, Beclin1 and ATG5, which are involved in the nucleation, elongation and maturation of the autophagosome and are commonly used indicators for assessing autophagic activities (Hurley and Young, 2017; Lee et al., 2018), suggesting induction of autophagy in these organs. In addition, a remarkable decrease in autophagy substrate p62, a prevalent marker for monitoring autophagic flux (Zhou et al., 2012), suggested CSFV-induced complete autophagy in infected organs exhibiting severer pathological injuries and higher viral loads. By contrast, no obvious autophagy was detectable in the infected organs with slighter pathological changes and lower viral loads, which implies that induction of autophagy during CSFV infection *in vivo* is to some extent tissue-specific and may be associated with CSFV replication *in vivo*. Taking into account the results obtained from ER stress detection, we suggest that CSFV infection differentially induces ER stress and autophagy in infected organs of pigs, which are probably associated with tissue injuries and tissue viral titers in these organs, and that there exists a potential link between CSFV-induced ER stress and autophagy.

ER stress-mediated autophagy has been well studied in the past several years (Ron and Walter, 2007; Song et al., 2018). Accumulating evidences have revealed that many viruses can trigger autophagy through the UPR signaling pathways (Yoshiyasu et al., 2013; Rashid et al., 2015; Lee et al., 2018), however, which UPR pathway is activated to mediate autophagic activities, and whether a cytoprotective autophagy or an autophagic cell death is induced upon ER stress, probably depend on the types of viruses and cells (Zhang and Wang, 2012; Parzych and Klionsky, 2013). As mentioned above, we have suggested a potential link between CSFV-induced ER stress and autophagy. To further confirm this relationship, we performed *in vitro* experiments and found that CSFV infection not only caused ER stress and activated UPR pathways, but also induced complete autophagy in cultured PK-15 and 3D4/2 cells, which were in line with our previous findings *in vitro* (Pei et al., 2014; He et al., 2017). However, there exist differences between the results obtained from the *in vivo* and *in vitro* experiments. Specifically, CSFV infection activated all three UPR pathways in infected organs with severe injury and only activated the IRE1 pathway in slightly injured organs, while cultured cells invoked the PERK and IRE1 pathways in response to CSFV infection at middle-late stage (24 hpi), which also can be evidenced by our previous study (He et al., 2017). After invading the body, the viruses face an integrated system which can not be simulated by any other cell models *in vitro*. This may help explain the reason for these discrepancies. Evidences also reveal that some viruses including DENV and HCV, can differentially regulate the UPR at different infection stages through a mechanism associated with differential expression of viral proteins, thus contributing to effective replication of viruses (Merquiol et al., 2011; Peña and Harris, 2011). Maybe, CSFV also activates UPR in a temporal manner, and the ATF-6 UPR pathway is probably suppressed to normal level by viral proteins at middle-late stage of CSFV infection for maintaining persist infection, which need further investigations. Anyway, our results showed that CSFV infection induces ER stress and autophagy *in vivo* and *in vitro*. We further found that pharmacological regulation of ER stress by ER stress agonists (TG) and inhibitors (4-PBA and TUDCA) remarkably changed the autophagic activities in CSFV-infected cells, suggesting that ER stress is required for CSFV-induced autophagy. Both 4-PBA and TUDCA are chemical chaperones that have been proven to mitigate ER stress through inhibition of PERK and IRE1 pathway (Uppala et al., 2017). In our study, TG treatment remarkably increased p-PERK, p-IRE1 and GRP78 expression as well as autophagic indicators in CSFV-infected cells, while the use of both 4-PBA and TUDCA effectively alleviate CSFV-induced ER stress and autophagy in cells through inhibiting PERK/eIF2α/ATF-4/CHOP and IRE1/GRP78 axis, suggesting the requirement of PERK and IRE1 signalings for CSFV-induced autophagy. We also found another BIP inducer X (Bix) robustly increased protein expression of GRP78 and autophagy markers (data not shown), further confirming the involvement of GRP78 in ER stress-mediated autophagy during CSFV infection. Taken together, our data suggested that CSFV infection induces ER stress-mediated autophagy *in vivo* and *in vitro*, probably *via* activation of the PERK and IRE1 pathways.

The roles of ER stress-mediated autophagy played in response to viruses belonging to the same *Flaviviridae* can be different. ER stress-mediated autophagy is exploited as a replication-favoring strategy by DENV and HCV (Dash et al., 2016; Lee et al., 2018), rather than by Japanese encephalitis virus, which induces autophagy through ER stress to negatively influence viral replication (Sharma et al., 2017). Considering this inconformity, we further investigated the effect of ER stress-mediated autophagy on CSFV replication *in vitro*, and found that treatment with ER stress agonists (TG) or autophagy inducer (RAPA) remarkably increased CSFV replication in cultured cells, however the replication of CSFV was significantly reduced in the cells incubated in the presence of ER stress inhibitors (4-PBA and TUDCA) or autophagy inhibitor (3-MA), indicating that CSFV-induced ER stress and autophagy are beneficial to its replication, which is consistent with our pervious findings (Pei et al., 2014; He et al., 2017). Since we have demonstrated that CSFV-induced autophagy is dependent on the ER stress (Figures 6–8), ER stress-mediated autophagy can be a potential mechanism for sustaining CSFV replication. To further confirm this relationship, we analyzed the effect of combination of ER stress and autophagy regulators on CSFV replication in cultured cells, and found that treatment with ER stress regulators significantly altered autophagy-regulated viral replication in CSFV-infected cells, suggesting that the replication of CSFV can be regulated by ER stress-induced autophagy in cultured cells. Similar results have been obtained in other studies, while the underlying mechanisms involved in ER stress-mediated autophagy are different. HCV activates autophagy through three UPR pathways, including PERK, IRE1 and ATF-6, to sustain viral replication in the infected hepatocytes (Dash et al., 2016). DENV-induced ER stress enhances autophagic activity and viral replication through the PERK and IRE1 signaling pathways both *in vitro* and *in vivo* (Lee et al., 2018). ER stress-mediated autophagy contributes to bluetongue virus infection *via* the PERK signaling pathway (Lv et al., 2015). From these findings, it can be seen that although cells can initiate the UPRs to relieve ER stress in response to virus infection, many viruses have evolved specific strategies to modulate the UPRs to sustain viral infection in host cells. Our preliminary findings also suggest the implication of the IRE1 and PERK signaling pathways in CSFV-induced autophagy and viral replication, but further confirmation is needed.

In conclusion, our findings confirm that CSFV can induce ER stress-mediated autophagy as a pro-survival mechanism to sustain viral replication *in vivo* and *in vitro*, which may be one of the potential strategies exploited by CSFV for immune evasion. Our findings will provide new insights into mechanisms of viral replication, persistent infection and pathogenesis of CSFV and promote development of new strategies for controlling CSF.

## Data Availability Statement

All datasets generated for this study are included in the article/Supplementary Material.

## Ethics Statement

The animal study was reviewed and approved by The Ethics Committee and the Laboratory Animal Care and Use Committee of South China Agricultural University.

## Author Contributions

EZ, JC, and MZ conceived the study. EZ, WC, YQ, SM, and SF performed the experiments. EZ, SM, JC, and MZ analyzed experimental results and data. WL, JF, KW, LY, and HD assisted with animal experiment. EZ wrote the manuscript. All authors read and approved the final manuscript.

### Conflict of Interest

The authors declare that the research was conducted in the absence of any commercial or financial relationships that could be construed as a potential conflict of interest.
